# Reflective functioning and emotion regulation in adolescents with a history of sexual offending: a comparative study with a non-offending control group

**DOI:** 10.1186/s13034-024-00802-3

**Published:** 2024-09-03

**Authors:** Mahdieh Pazhooyan, Fahimeh Fathali Lavasani, Zohreh Edalati Shateri, Komeil Zahedi Tajrishi

**Affiliations:** 1 Department of Clinical Psychology, School of Behavioral Sciences and Mental Health (Tehran Institute of Psychiatry), Iran University of Medical Sciences, Tehran, Iran; 2 Faculty of Education Sciences and Psychology, Ferdowsi University of Mashhad, Mashhad, Iran

**Keywords:** Adolescent sexual offending, Reflective functioning, Emotion regulation strategies, Emotion dysregulation

## Abstract

**Background:**

Sexual offenses encompass a diverse array of behaviors across various contexts, affecting numerous individuals. Despite the prevalence of sexual offending among adolescents, there is still a limited understanding of this population. To contribute further to the literature in this field, the present study was conducted to compare a group of male adolescents convicted of sexual offenses with a control group in terms of reflective functioning (RF), emotion regulation (ER) strategies, and emotion dysregulation (ED).

**Methods:**

60 male adolescents aged 12 to 18 years (M = 16.90; *SD* = 0.97) who had been convicted of at least one serious sexual offense were recruited from male adolescents referred by juvenile courts to the Legal Medicine Organization in Mashhad, Iran, and compared with a control group of non-offending adolescents consisting of 60 male adolescents aged 12 to 18 years (M = 16.97; *SD* = 0.82) who were attending school. The groups were matched on age and education level.

**Results:**

A comparison between these two groups revealed that adolescents with a history of sexual offending exhibited poorer RF capacity, greater use of suppression as an ER strategy, and higher scores in all ED domains (*p’s* < 0.001) except non-acceptance of emotional responses compared with the control group.

**Conclusions:**

Results suggest that RF, ER strategies, and ED need to be considered as important psychological factors in understanding and treating adolescents with a history of sexual offending.

## Introduction

Sexual offenses entail a broad range of behaviors in various situations that victimize a multitude of individuals [1]. In 2014, adolescents accounted for 21% of all arrests for sexual offenses in the United States [2]. Rates of sexual offenses committed by juveniles also vary from 5 to 24% in Europe [3]. Despite being a significant public health issue, research on sexual offending has focused predominantly on adult offenders. Consequently, there is a limited understanding of adolescent sexual offending, leaving a critical gap in the literature that needs to be addressed [4]. Paying attention to these youth is essential, as studies have found that adolescents who engage in sexually abusive behavior constitute a distinct group from adults who sexually offend, differing in their risk factors [5], and having a lower risk of reoffending sexually [6]. According to a previous research, these adolescents appear to be more prone to non-sexual recidivism [7].

Given the multidimensional nature of sexual offending, a wide range of risk factors, including developmental, biological, societal, familial, and psychological factors have been explored to enhance our understanding of sexual offending and aid in developing more effective assessment and treatment programs for individuals who commit sexual offenses [1, 6]. In terms of psychological factors, the literature suggests that deficits in reflective functioning (RF; [8]) and emotion regulation (ER; [9]) could contribute to sexual offending. However, most studies highlighting the role of these variables in sexual offending are derived from samples of adult offenders, and their generalizability to adolescents who have sexually offended remains uncertain. Therefore, there is a need for studies that examine these psychological variables specifically in the context of adolescents involved in sexual offending. In the following sections, we will delve into the existing literature that explores the complex relationships between these variables and sexual offending. Through this analysis, we aim to underscore a critical gap in the current literature concerning these variables, particularly in relation to young people who have been involved in sexual offending.

## Reflective functioning and sexual offending

Mentalization is a particular dimension of social cognition that explains how people make sense of their social world by envisioning their mental states and those of others (e.g., feelings, beliefs, intentions, and desires) to make sense of their social world [10]. The concept of mentalization is multidimensional and overlaps with several constructs, such as theory of mind (ToM), mindfulness, perspective-taking, and empathy [11]. The term RF describes the psychological processes that underlie the ability to mentalize and provides a framework for assessing mentalization [12].

It has been found that lower levels of RF prevail to varying degrees in both adult [13] and young [14] samples of offenders which is manifested through a detachment from the needs and feelings of others or an inability to take into account how one’s actions affect other people [15]. Levinson and Fonagy [13] demonstrated that the RF of adult prisoners with violent offenses was significantly lower than that of those who had committed non-violent offenses or individuals with personality disorders. Another study also reported that compared with non-offending adolescents, juvenile offenders had lower RF scores [16]. These findings support the hypothesis that the inability to reflect on one’s mental state and that of others may play an important role in criminal behavior.

Research has also indicated a possible link between RF impairments and sexual offending. According to Fonagy [17], a lack of RF capacity may lead to the development of violent behavior, including sexual violence, by reducing empathy and a sense of responsibility for actions, enabling the treatment of others as physical objects and facilitating the development of cognitive distortions related to unacceptable conduct. In addition, there is ample indirect evidence about associations of deficits in other mentalizing-related capacities, such as ToM, empathy, and perspective-taking with sexual offending [18, 19]. For instance, Ward et al. [20] suggested that deficits in ToM are likely to lead to psychological problems including intimacy deficits, difficulties with empathy, and cognitive distortions, all of which have been identified as core features of individuals who commit sexual offenses.

Despite the established conceptual links between RF impairment and sexual offending, to the best of our knowledge, only one study has examined RF in juveniles with a history of sexual offending. Using a multiple-case design, this study found significant difficulties in perspective-taking, RF, and empathy in eight adolescents who engaged in harmful sexual behavior [21]. Although findings from this study suggest that RF may be a relevant construct in the context of sexual offending in adolescents, the small sample size, the lack of inclusion of a control group of non-offending adolescents, and the non-use of appropriate and valid measures of RF are among the limitations of this study that should be addressed in future studies.

### Emotion regulation and sexual offending

Research has consistently shown that sexual offenders typically have problems with self-regulation [22], especially ER [9, 23]. Many individuals who sexually offend may struggle to differentiate, recognize, and express their emotions, often resorting to maladaptive strategies to suppress negative emotions [24]. Such ER difficulties can impair their ability to control their impulses when faced with sexual arousal, increasing the likelihood of using coercive means for sexual gratification [25]. In line with this, treatments such as multiple-family group intervention have been developed to reduce maladaptive emotion regulation in male adolescents incarcerated for sexual offenses [26].

The concept of ER reflects processes by which individuals employ a variety of strategies to influence what emotions they have, when they experience them, and how those emotions are experienced and expressed [27]. The process model of ER distinguishes ER strategies according to the timing of their main influence on the emotion-generating process, with a focus on cognitive reappraisal and expressive suppression. Cognitive reappraisal is an antecedent-focused strategy in which a potentially emotionally charged situation is interpreted in a non-emotional way. In contrast, expressive suppression is a response-focused strategy in which ongoing emotional-expressive behavior is inhibited. When analyzing these two forms of ER strategies, it is repeatedly shown that reappraisal is preferable to suppression as the latter is associated with reduced manifestation and experience of positive and negative emotions [28]. In addition, chronic and rigid use of expressive suppression appears to be a trigger for violence and aggressive behavior [29]. Regarding the relationship between the extent of use of these two ER strategies and sexual offending, Gillespie et al. [30] reported that adults who sexually offended showed no significant differences in the use of these specific strategies (i.e., cognitive reappraisal and expressive suppression) compared to the community sample. However, the sample of this study included adults with a history of sexual offending, which limits the generalizability of the results to adolescent population.

While certain conceptualizations of ER place a strong emphasis on the employment of ER strategies for reducing emotional arousal and controlling emotional experience and expression [31], others suggest that ER may be conceptualized as involving (a) awareness and understanding of emotions, (b) accepting emotional responses, (c) being able to control one’s impulses and participate in goal-directed behaviors when feeling negative emotions, and (d) the flexibility to employ situationally appropriate ER strategies to adjust emotional responses as needed to fulfill personal goals and situational demands. The relative presence of deficits in one or more of these domains would indicate difficulties in ER, or emotion dysregulation (ED;[32]). Research suggests that the risk of recidivism in certain types of crimes among juveniles is related to ED [33].

ED has also been recognized as highly problematic in adults who have committed sexual offenses [34]. However, to the best of our knowledge, only one study has examined ED in adolescents with a history of sexual offending [35], and its findings diverge from those observed in adults [30]. In this sole study, Jones et al. [35] reported that adolescents who had committed sexual offenses exhibited greater difficulties in all ER domains, except awareness and acceptance of emotions, compared to non-offending controls. Conversely, Gillespie et al. [30] found that sexually offending adults reported significantly more impairments in the acceptance of emotional responses compared to a community sample. Taken together, these findings highlight the need for more studies to comprehensively examine ED among adolescents who have committed sexual offenses compared to non-offending juveniles. Further research is also essential to clarify which specific ED domains are pertinent to adolescent sexual offending.

### The present study

In the present study, we aimed to examine differences in RF, ER strategies, and ED in a group of male adolescents with a history of sexual offending and a comparison group of non-offending adolescents from the general population. In terms of RF, existing theoretical frameworks suggest that adults with sexual offenses tend to have lower levels of RF [8]. However, due to the limited literature on this topic on adolescents with a history of sexual offending, no definitive hypothesis was made regarding possible differences in RF between the two groups. Furthermore, previous studies with adult offenders found no significant differences between males with a history of sexual offending and control subjects in terms of the use of different ER strategies [30]. However, given the potential disparities between the adolescent and adult samples described above, no definitive hypotheses were made regarding possible differences in the use of cognitive reappraisal and/or expressive suppression as ER strategies between the groups. Concerning ED, consistent with previous research [35], it was hypothesized that adolescents with a history of sexual offending would exhibit elevated levels of ED compared to controls. Nevertheless, no solid hypotheses were formulated regarding possible differences in specific ED domains.

## Method

### Participants and procedure

This cross-sectional comparative study consisted of 120 male adolescents (aged 12 to 18 years) who were divided into two groups: Adolescents with a history of sexual offending and a control group of non-offending adolescents. The sexual offending sample was recruited from a population of adolescents who were referred for mental health evaluation to the Legal Medicine Organization in Mashhad by juvenile courts from July 2021 to June 2022. Based on their judicial record, 74 adolescents who had been convicted of at least one serious sexual offense, including rape and molestation of a victim of any age, were invited to participate in the study. Fourteen adolescents declined our invitation. Consequently, data was collected from 60 male adolescents (Mean age = 16.90; *SD* = 0.97) who had committed sexual offenses within one year prior to participation in the study. All participants were living with their families under court supervision at the time of data collection.

For the control group of non-offending adolescents, 60 male adolescents (Mean age = 16.97; *SD* = 0.82) who had no history of any criminal activity were recruited from public school registries in Mashhad city. The exclusion criteria for both of these groups were significant intellectual and developmental disabilities, inability to read above a fifth-grade reading level, and a clinical history of severe mental disorders such as schizophrenia and psychosis-related disorders and affective disorders, as assessed by court reports (offender sample) and psychiatric records (control sample). The two groups were matched in terms of age and education.

For data collection from the sexual offending sample, eligible participants were invited to attend an appointment with their legal guardians (one of their parents) where they completed consent forms and questionnaires. Before participation, they signed consent forms after receiving information about the study’s objectives and the voluntary and confidential nature of their involvement. The data collection process was supervised by a clinical psychologist serving as the lead investigator. Similar procedures were employed for collecting data from school-attending adolescents, except they completed the measures in their classrooms under the supervision of a specially trained research assistant. The study received formal approval from the ethics review board of the Iran University of Medical Sciences (IR.IUMS.REC.1400.214).

### Measures^1^

#### Reflective functioning questionnaire for Youth (RFQ-Y)

The RFQ-Y [36] is a 46-item self-report questionnaire that measures RF in adolescents. In this questionnaire, adolescents are asked to rate statements about their understanding of their own and others’ mental states on a six-point Likert scale ranging from 1 (*“strongly disagree”*) to 6 (*“strongly agree”*). The answers are then summed up to compute a total score, with higher scores indicating a greater RF capacity. A preliminary study by Esmaeilinasab et al. (2024) has demonstrated promising psychometric properties of the Persian version of the RFQ-Y in adolescents.

#### Emotion regulation questionnaire for children and adolescents (ERQ–CA)

The ERQ-CA [37] is a revised version of the self-report Emotion Regulation Questionnaire (ERQ;[31])which evaluates the ER strategies of cognitive reappraisal and expressive suppression in children and adolescents. It comprises 10 items rated on a five-point Likert scale ranging from 1 (*“strongly disagree”*) to 5 (*“strongly agree”*). Regarding the validity and reliability of its subscales, the Persian version of the ERQ-CA has demonstrated strong psychometric qualities [38].

#### Difficulties in emotion regulation scale (DERS)

The DERS [32] is a 36-item measure that assesses difficulties in ER. The items of this instrument are rated on a five-point Likert scale ranging from 1 (*“almost never”*) to 5 (*“almost always”*), comprising six subscales: lack of emotional awareness (Awareness), poor emotional clarity (Clarity), non-acceptance of emotional responses (Non-acceptance), impulsive control difficulties (Impulse), difficulties engaging in goal-directed behaviors (Goals), and limited access to emotion regulation strategies (Strategies). Higher scores indicate greater difficulties in the ER. In the present study, we administered the Persian version of the DERS, which has shown good internal consistency and validity in previous research with Iranian adolescents [39].

### Data analyses

In the present study, SPSS 20 software was used for data entry and statistical analyses. The normality of the distribution of the study variables was assessed using the Kolmogorov–Smirnov test which indicated that the data followed a normal distribution (*p* >.05). One-way analysis of variance (ANOVA) and multivariate analysis of variance (MANOVA) were then employed to compare the two groups on the study variables, while partial eta squared (*η*_*p*_^*2*^) values of 0.01, 0.06, and 0.14 were interpreted as small, medium, and large effects, respectively. Given the numerous group comparison tests, the alpha was set at 0.001 to balance type I and type II errors.

## Results

Table 1 shows that the groups did not differ significantly in demographic variables, including age and education level (*p* >.05). Descriptive statistics for all the study variables are presented in Table 2. One-way analysis of variance (ANOVA) was first employed to scrutinize group differences in the RFQ-Y total score. The results revealed significantly lower scores in the group with a history of sexual offending than in the control group [F (1, 118) = 31.87, *p* <.001, *η*_*p*_^*2*^ = 0.21]. Regarding the ERQ-CA scores, the MANOVA test indicated a significant influence of the composite dependent variable, comprising the ERQ-CA Cognitive Reappraisal and Expressive Suppression subscales [Wilks’ Lambda = 0.85, F (1, 118) = 10.61, *p* <.001, *η*_*p*_^*2*^ = 0.15]. However, univariate tests revealed a significant effect of group only on the ERQ-CA Expressive Suppression subscale [F (1, 118) = 15.80, *p* <.001, *η*_*p*_^*2*^ = 0.12]. Furthermore, a MANOVA test was conducted to explore group differences in DERS scores. The multivariate tests showed a significant effect of the group variable on the composite dependent variable (i.e., the DERS subscales; Wilks’ Lambda = 0.71, F (1, 118) = 7.53, *p* <.001, *η*_*p*_^*2*^ = 0.29.) As detailed in Table 2, univariate tests further revealed significant effects of the group variable on the DERS subscales of Goals [F (1, 118) = 22.63, *p* <.001, *η*_*p*_^*2*^ = 0.16], Impulse [F (1, 118) = 28.89, *p* <.001, *η*_*p*_^*2*^ = 0.20], Awareness [F (1, 118) = 11.67, *p* <.001, *η*_*p*_^*2*^ = 0.09], Strategies [F (1, 118) = 12.33, *p* <.001, *η*_*p*_^*2*^ = 0.09], and Clarity [F (1, 118) = 17.56, *p* <.001, *η*_*p*_^*2*^ = 0.13].

**Table 1:** The comparison of groups in age and education

Variable	Group		Group Comparisons	
	NO (*n* = 60) Mean (SD)	SO (*n* = 60) Mean (SD)		
			t/Χ^2^	*p*
Age	16.97 (0.82)	16.90 (0.97)	0.42	0.67
Education (%)			3.36	0.34
Grade 9	6 (10.00)	6 (10.00)		
Grade 10	3 (5.00)	3 (5.00)		
Grade 11	36 (60.00)	27 (45.00)		
Grade 12	15 (25.00)	24 (40.00)		

Note. *No* = non-offending adolescents; *SO* = adolescents who committed sexual offenses; *SD* = Standard Deviation

**Table 2:** Descriptive statistics for study variables and group comparisons

Measures		Groups						Group comparisons			Observed power
		NO (*n* = 60)			SO (*n* = 60)						
		Mean (*SD*)	*α*	*MIC*	Mean (*SD*)	*α*	*MIC*	F	*p*	*η*_*p*_^*2*^	
RFQ-Y	Total score	7.09 (1.15)	0.86	0.21	6.21 (0.39)	0.75	0.11	31.87	< 0.001	0.21	1.00
ERQ-CA											
	Cognitive Reappraisal	20.15 (3.06)	0.72	0.32	18.75 (3.50)	0.75	0.34	5.44	0.02	0.04	0.64
	Expressive Suppression	11.95 (3.03)	0.66	0.25	13.98 (2.55)	0.69	0.27	15.81	< 0.001	0.12	0.98
DERS											
	Goals	13.57 (4.39)	0.77	0.41	17.18 (3.93)	0.76	0.40	22.63	< 0.001	0.16	1.00
	Impulse	13.07 (4.29)	0.81	0.44	17.42 (4.57)	0.75	0.33	28.89	< 0.001	0.20	1.00
	Non-acceptance	12.93 (4.71)	0.67	0.26	15.23 (5.01)	0.73	0.23	6.72	0.011	0.05	0.73
	Awareness	15.17 (4.20)	0.66	0.25	17.93 (4.66)	0.72	0.30	11.67	< 0.001	0.09	0.92
	Strategies	16.13 (5.42)	0.77	0.31	19.27 (4.29)	0.79	0.29	12.33	< 0.001	0.09	0.94
	Clarity	10.08 (3.84)	0.82	0.48	13.20 (4.29)	0.75	0.38	17.56	< 0.001	0.13	0.99

*Note. NO* = non-offending adolescents; *SO* = adolescents who committed sexual offenses; *SD* = Standard deviation; *RFQ-Y =* Reflective Function Questionnaire for Youth; *DERS* = Difficulties in Emotion Regulation Scale; *ERQ-CA* = Emotion Regulation Questionnaire for Children and Adolescents; *α* = Chrobach’s Alpha; *MIC* = Mean Inter-Item Correlation

## Discussion

This study aimed to examine differences in RF, ER strategies, and ED between adolescents who committed sexual offenses and a control group of non-offenders. Overall, adolescents with a history of sexual offending showed lower levels of RF, higher use of expressive suppression as an ER strategy, and greater ED compared with those in the control group.

The significant difference between the RF scores of adolescents with sexual offenses and those of the non-offender sample suggests that these youth may have limited capacity to think about or “mentalize” their own mental states or the mental states of others in terms of feelings, thoughts, beliefs, or motivation. These findings are comparable to those from a sample of adolescent offenders with harmful sexual behavior who showed significant problems with RF [21]. Greater deficits in RF have also been observed in male juvenile offenders compared to non-offending adolescents [16]. Keenan and Ward [40] argued that difficulties in understanding and attributing different types of mental states to other people are likely to lead to a lack of empathy and could create an interpersonal context in which inappropriate and potentially abusive behaviors such as sexual assault could occur. Taken together, these findings are in line with Allen et al.’s [41] assumption that “mind-blindness”, defined as the inhibition of mentalizing, allows one to distance oneself from the victim, thus facilitating the acting out of one’s aggressive impulses.

In terms of ER strategies, the findings of this study showed that adolescents who had sexually offended scored higher on expressive suppression than the non-offender sample. These results appear to contrast with those of Gillespie et al. [30], who found no significant differences between different groups of adults who had committed sexual, violent, or homicide offenses, and a community sample in terms of cognitive reappraisal and expressive suppression. One plausible explanation for this controversial pattern of findings is that conclusions derived from studies of adults who have sexually offended may not be generalizable to explain sexually abusive behavior in adolescents [42]. Our findings are in line with those of Habib et al. [15], who reported that incarcerated males were more likely to use expressive suppression than normative samples from other studies in Lebanon and the Arab region. There is also evidence that the inflexible use of expressive suppression may contribute to aggressive behavior by increasing negative affect, reducing the individual’s inhibition against aggression, impairing decision-making processes through the depletion of cognitive resources, decreasing social networks, increasing physiological arousal, and making it more difficult to cope with challenging situations [43]. When considering the other ER strategy, we found no significant differences between the two groups in terms of cognitive reappraisal, suggesting that adolescents who had committed sexual offenses did not have problems in reappraising their emotions compared to the non-offender control group. This non-significant result is consistent with the conclusions of a previous study, which found no differences between juveniles who had committed sexual offenses and the control group in terms of reappraisal abilities [35]. However, given the dearth of research on specific ER strategies in sexually offending adolescents, further studies are needed to replicate these findings.

Our results regarding ED showed significant differences between the two groups in all domains of ED except non-acceptance of emotional responses. In other words, adolescents who had sexually offended reported greater difficulties in being aware of and understanding their emotions, engaging in goal-directed behaviors and controlling impulsive behaviors when experiencing negative emotions, and using situationally appropriate ER strategies flexibly compared to those in the non-offending sample. Previous work suggests that men who sexually offend are often characterized by deficits in ER [44]. However, there is a paucity of research that has comprehensively assessed ED in adolescents charged with sexual crimes. Our findings support previous research [35], demonstrating that ED may represent a characteristic feature of adolescents who commit sexual offenses. This assertion is supported by Moriarty et al. [45] who also found that adolescents who become sexually delinquent exhibit deficits in emotional understanding and are less able to repair unpleasant moods and prolong positive moods. The findings from this study seem to be inconsistent with those of a previous study that found evidence of impairments in the acceptance of emotional responses in adult males who had committed sexual offenses [30]. It is possible that the use of an adult sample in the latter study may explain these contradictory findings. Nonetheless, our findings suggest that ED may serve as a significant variable in the context of sexual offending among adolescents.

The findings from the current study should be interpreted within the context of several limitations. First, the use of self-report measures for data collection may lead to shared method variance, potentially influencing our results. Thus, future studies should employ multi-informant approaches in data gathering to enhance the robustness of findings. Second, the Expressive Suppression subscale of the ERQ-CA and the Non-acceptance and Awareness subscales of the DERS did not demonstrate acceptable alpha coefficients. This may be attributed to the limited number of items in these subscales, as Cronbach’s alpha is sensitive to item count. However, the MIC provides a more reliable indicator in these cases, as it is less affected by the length of the scales. Future research should examine whether these findings are unique to this study or if they generalize to other populations and settings. Third, our study utilized a cross-sectional design which precludes causal inferences. Longitudinal studies are needed to elucidate the causal relationships between the variables examined in this study and adolescent sexual offending. Further, the sample size in our study was relatively small. Future research efforts could strengthen the generalizability and reliability of our findings by including larger sample sizes. Finally, another limitation of this study is the lack of consideration of additional variables that could moderate the observed differences. Future studies should incorporate variables such as the age of the victims, reoffender versus non-reoffender status, relationships to the victims (known versus unknown), and offender typologies (sex-only or specialists versus sex-plus or generalist offenders). Including these variables would provide a more nuanced understanding of the factors distinguishing different types of adolescents who sexually offend.

## Conclusion

This study builds upon previous research by providing new insights into the reflective functioning capacity and emotion regulation abilities of adolescents who have committed sexual offenses. In short, our findings indicate that adolescents with a history of sexual offending appear to be characterized by lower levels of RF, higher use of suppression as an ER strategy, and greater difficulties in ER compared to non-offending controls. This knowledge adds important information to the growing body of research on adolescent sexual offending. These findings also suggest that adolescents who commit sexual offenses may benefit from mentalization and emotion regulation-based interventions aimed at improving RF and enhancing ER skills. Therefore, findings from this study may encourage future studies to examine whether incorporating such therapeutic approaches into intervention programs can effectively reduce the risk of sexual recidivism in adolescents.

## Data Availability

The datasets generated during and/or analyzed during the current study are available from the corresponding author upon reasonable request.

## Abbreviations

RF: Reflective Functioning
ER: Emotion Regulation
ED: Emotion Dysregulation

## Footnotes

1 The internal consistency of measures was evaluated through Cronbach’s alpha (*α* ≥ 0.70 considered acceptable; Cheung & Rensvold, 2002) and mean inter-item-correlation (MIC; 0.15 ≤ MICs ≤ 0.50 considered adequate; Clark & Watson, 1995). The results are shown in Table 2.

## References

[1] Ryan G, Leversee TF, Lane S. Juvenile sexual offending: Causes, consequences, and correction. Wiley; 2011.

[2] Federal Bureau of Investigation. Crime in the United States 2014 [ https://www.fbi.gov/about-us/cjis/ucr/crime-in-the-u.s/2014/crime-in-the-u.s.-2014/tables/table-39

[3] Margari F, Lecce PA, Craig F, Lafortezza E, Lisi A, Pinto F, et al. Juvenile sex offenders: personality profile, coping styles and parental care. Psychiatry Res. 2015;229(1):82–8. 26233829 10.1016/j.psychres.2015.07.066

[4] Siria S, Echeburúa E, Amor PJ. Characteristics and risk factors in juvenile sexual offenders. Psicothema. 2020;32(3):314–21. 32711665 10.7334/psicothema2019.349

[5] Lussier P, Blokland A, Mathesius J, Pardini D, Loeber R. The childhood risk factors of adolescent-onset and adult-onset of sex offending: Evidence from a prospective longitudinal study. Sex offenders: A criminal career approach. Wiley-Blackwell; 2015. p. 93–128.

[6] Ryan EP. Juvenile sex offenders. Child Adolesc Psychiatr Clin N Am. 2016;25(1):81–97. 26593121 10.1016/j.chc.2015.08.010

[7] Fanniff AM, Schubert CA, Mulvey EP, Iselin AR, Piquero AR. Risk and outcomes: are adolescents charged with sex offenses different from other adolescent offenders? J Youth Adolesc. 2017;46(7):1394–423. 27406040 10.1007/s10964-016-0536-9

[8] Baker E, Beech A, Tyson M. Attachment disorganization and its relevance to sexual offending. J Family Violence. 2006;21(3):221–31. 10.1007/s10896-006-9017-3

[9] Gunst E, Watson JC, Desmet M, Willemsen J. Affect regulation as a factor in sex offenders. Aggress Violent Beh. 2017;37:210–9. 10.1016/j.avb.2017.10.007

[10] Fonagy P, Luyten P. A developmental, mentalization-based approach to the understanding and treatment of borderline personality disorder. Dev Psychopathol. 2009;21(4):1355–81. 19825272 10.1017/S0954579409990198

[11] Luyten P, Campbell C, Allison E, Fonagy P. The Mentalizing Approach to Psychopathology: state of the art and future directions. Annu Rev Clin Psychol. 2020;16:297–325. 32023093 10.1146/annurev-clinpsy-071919-015355

[12] Fonagy P, Target M, Steele H, Steele M. Reflective-functioning manual version 5 for application to adult attachment interviews. 1998.

[13] Levinson A, Fonagy P. Offending and attachment: the relationship between interpersonal awareness and Offending in a Prison Population with Psychiatric Disorder. Can J Psychoanal / Revue Canadienne De Psychanalyse. 2004;12(2):225–51.

[14] Möller C, Falkenström F, Holmqvist Larsson M, Holmqvist R. Mentalizing in young offenders. Psychoanal Psychol. 2014;31(1):84–99. 10.1037/a0035555

[15] Abi-Habib R, Wehbe N, Badr K, Tohme P. Do prisoners mentalize differently? Investigating attachment and reflective functioning in a sample of incarcerated Lebanese men. Int J Forensic Mental Health. 2020;19(2):183–97. 10.1080/14999013.2019.1684403

[16] Protic S, Wittmann L, Taubner S, Dimitrijevic A. Differences in attachment dimensions and reflective functioning between traumatized juvenile offenders and maltreated non-delinquent adolescents from care services. Child Abuse Negl. 2020;103:104420. 32146268 10.1016/j.chiabu.2020.104420

[17] Fonagy P. Male perpetrators of violence against women: an attachment theory perspective. J Appl Psychoanal Stud. 1999;1(1):7–27. 10.1023/A:1023074023087

[18] Barnett GD, Mann RE. Cognition, Empathy, and sexual Offending. Trauma Violence Abuse. 2013;14(1):22–33. 23258800 10.1177/1524838012467857

[19] Elsegood KJ, Duff SC. Theory of mind in men who have sexually offended against children: a U.K. comparison study between child sex offenders and nonoffender controls. Sex Abuse: J Res Treat. 2010;22(1):112–31. 10.1177/1079063209359926 20133963

[20] Ward T, Keenan T, Hudson SM. Understanding cognitive, affective, and intimacy deficits in sexual offenders: a developmental perspective. Aggress Violent Beh. 2000;5(1):41–62. 10.1016/S1359-1789(98)00025-1

[21] Zaniewski B, Dallos R, Stedmon J, Welbourne P. An exploration of attachment and trauma in young men who have engaged in harmful sexual behaviours. J Sex Aggress. 2019;26:1–17.

[22] Stinson J, Sales B, Becker J. Multimodal self-regulation theory: A new integration. 2008. pp. 167–202.

[23] Howells K, Day A, Wright S. Affect, emotions and sex offending. Psychol Crime Law - PSYCHOL CRIME LAW. 2004;10:179–95. 10.1080/10683160310001609988

[24] Byrne G, Bogue J, Egan R, Lonergan E. Identifying and describing emotions: measuring the effectiveness of a brief, Alexithymia-Specific, intervention for a sex Offender Population. Sex Abuse. 2016;28(7):599–619. 25420556 10.1177/1079063214558940

[25] Craig AN, Peterson ZD, Janssen E, Goodrich D, Heiman JR. The impact of sexual arousal and emotion regulation on men’s sexual aggression proclivity. J Interpers Violence. 2022;37(1–2):NP264–80. 32345118 10.1177/0886260520915544

[26] Keiley MK, Zaremba-Morgan A, Datubo-Brown C, Pyle R, Cox M. Multiple-Family Group Intervention for Incarcerated Male adolescents who sexually offend and their families: change in maladaptive emotion regulation predicts adaptive change in adolescent behaviors. J Marital Fam Ther. 2015;41(3):324–39. 24809985 10.1111/jmft.12078

[27] Gross JJ. The emerging field of emotion regulation: an integrative review. Rev Gen Psychol. 1998;2(3):271–99. 10.1037/1089-2680.2.3.271

[28] Gross JJ. Emotion regulation: affective, cognitive, and social consequences. Psychophysiology. 2002;39(3):281–91. 12212647 10.1017/S0048577201393198

[29] Roberton T, Daffern M, Bucks RS. Beyond anger control: Difficulty attending to emotions also predicts aggression in offenders. Psychol Violence. 2015;5(1):74–83. 10.1037/a0037214

[30] Gillespie SM, Garofalo C, Velotti P. Emotion regulation, mindfulness, and alexithymia: specific or general impairments in sexual, violent, and homicide offenders? J Criminal Justice. 2018;58:56–66. 10.1016/j.jcrimjus.2018.07.006

[31] Gross JJ, John OP. Individual differences in two emotion regulation processes: implications for affect, relationships, and well-being. J Pers Soc Psychol. 2003;85(2):348–62. 12916575 10.1037/0022-3514.85.2.348

[32] Gratz KL, Roemer L. Multidimensional Assessment of emotion regulation and dysregulation: development, factor structure, and initial validation of the difficulties in emotion regulation scale. J Psychopathol Behav Assess. 2004;26(1):41–54. 10.1023/B:JOBA.0000007455.08539.94

[33] Salinas KZ, Venta A. Testing the role of emotion dysregulation as a predictor of Juvenile Recidivism. Eur J Investig Health Psychol Educ. 2021;11(1):83–95. 34542451 10.3390/ejihpe11010007 PMC8314338

[34] Gunst E, Watson J, Willemsen J, Desmet M, Loeys T, Vanhooren S. A quest for self-soothing: a systematic case study into emotion-focused therapy with an emotionally avoidant client who committed sexual offenses. J Clin Psychol. 2020;76(4):676–87. 31777086 10.1002/jclp.22906

[35] Jones S, Joyal CC, Cisler JM, Bai S. Exploring emotion regulation in Juveniles who have sexually offended: an fMRI study. J Child Sex Abus. 2017;26(1):40–57. 27997290 10.1080/10538712.2016.1259280

[36] Sharp C, Williams LL, Ha C, Baumgardner J, Michonski J, Seals R, et al. The development of a mentalization-based outcomes and research protocol for an adolescent inpatient unit. Bull Menninger Clin. 2009;73(4):311–38. 20025427 10.1521/bumc.2009.73.4.311

[37] Gullone E, Taffe J. The emotion regulation questionnaire for children and adolescents (ERQ-CA): a psychometric evaluation. Psychol Assess. 2011;24:409–17. 22023559 10.1037/a0025777

[38] Lotfi M, Bahrampouri L, Amini M, Fatemitabar R, Birashk B, Entezari M, et al. Persian adaptation of emotion regulation questionnaire for children and adolescents (ERQ-CA). J Mazandaran Univ Med Sci. 2019;29(175):117–28.

[39] Vafa Z, Azizi M, Elhami Athar M. Predicting Academic Alienation from emotion dysregulation, social competence, and peer relationships in School-Attending girls: a multiple-regression Approach. Front Psychol. 2021;12:755952. 35035367 10.3389/fpsyg.2021.755952 PMC8759297

[40] Keenan T, Ward T. A theory of mind perspective on cognitive, affective, and intimacy deficits in child sexual offenders. Sex Abuse. 2000;12:49–60. 10729959 10.1177/107906320001200106

[41] Allen JG, Fonagy P, Bateman AW. Mentalizing in clinical practice. American Psychiatric Pub; 2008.

[42] Puszkiewicz KL, Stinson JD. Pathways to delinquent and sex offending behavior: the role of childhood adversity and environmental context in a treatment sample of male adolescents. Child Abuse Negl. 2019;98:104184. 31675528 10.1016/j.chiabu.2019.104184

[43] Roberton T, Daffern M, Bucks R. Emotion regulation and aggression. Aggress Violent Beh. 2012;17:72–82. 10.1016/j.avb.2011.09.006

[44] Gillespie SM, Mitchell IJ, Fisher D, Beech AR. Treating disturbed emotional regulation in sexual offenders: the potential applications of mindful self-regulation and controlled breathing techniques. Aggress Violent Beh. 2012;17(4):333–43. 10.1016/j.avb.2012.03.005

[45] Moriarty N, Stough C, Tidmarsh P, Eger D, Dennison S. Deficits in emotional intelligence underlying adolescent sex offending. J Adolesc. 2001;24(6):743–51. 11790054 10.1006/jado.2001.0441

